# Mycobacterium abscessus Ventriculoperitoneal Shunt Infection

**DOI:** 10.7759/cureus.16356

**Published:** 2021-07-13

**Authors:** Danay Herrera, Aunie Danyalian, Daniel Kaswan, Nicole Cohen, Miguel Edelstein, Andres Rivero, Paola R Solari

**Affiliations:** 1 Internal Medicine, Aventura Hospital and Medical Center, Aventura, USA; 2 Infectious Disease, Aventura Hospital and Medical Center, Aventura, USA; 3 Infectious Disease, Aventura Hospital, Miami, USA; 4 Infectious Disease, Aventura Hospital and Medical Center, Miami, USA

**Keywords:** mycobacterium abscessus, non-tuberculous mycobacteria, cns infections, ventricular catheter, rgm

## Abstract

*Mycobacterium abscessus* is a rapidly growing mycobacterium (RGM) known to be abundant in soil, dust and water. Unlike other non-tuberculous mycobacteria, RGM is typically resistant to first-line anti-tuberculosis drugs. These organisms are known to cause community and hospital-acquired infections; however, central nervous system (CNS) infections caused by these pathogens have not been abundantly reported. As the use of vascular catheters and prosthetic devices is becoming more common, a rise in CNS infections secondary to *M. abscessus *has been noted. Here, we present such a case where the removal of a ventricular catheter was necessary to guarantee source control and eradication of the infection.

## Introduction

*Mycobacterium abscessus* is a rapidly growing mycobacterium (RGM) known to be abundant in soil, dust and water. Unlike other non-tuberculous mycobacteria, RGM is typically resistant to first-line anti-tuberculosis drugs. While it has emerged as the cause of an increasing number of community and hospital-acquired infections in people, central nervous system (CNS) infections caused by the organism are uncommon and have not been widely reported [1-5]. The pathogen has previously been associated with skin and soft tissue infections, as well as with pulmonary disease in immunocompromised individuals. With the increased use of vascular catheters and prosthetic devices; however, these organisms are now responsible for more invasive diseases [4]. In this report, we present a case of CNS infection secondary to *M. abscessus* involving a ventriculoperitoneal (VP) shunt requiring the removal of the ventricular catheter to ensure control and eradication of the infection.

## Case presentation

The patient is a 97-year-old Caucasian female with a past medical history pertinent for dementia and normal pressure hydrocephalus with a right-sided ventriculo-peritoneal shunt placed over a decade prior to admission who presented to our facility for swelling and erythema over the right side of her neck. General physical examination demonstrated non-tender swelling and erythema over the right side of the patient's neck with no pain on movement. On admission, the patient’s complete blood cell count was within normal limits and she was afebrile. The patient was taken for revision of the shunt and intraoperative findings were consistent with signs of a disrupted shunt. The patient tolerated the procedure well and was discharged home. Cultures taken from shunt aspirate did not yield any microorganisms. Cerebrospinal fluid (CSF) analysis did not demonstrate white blood cells, red blood cells and glucose was normal at 68 mg/dL. The patient was readmitted for drainage from the surgical site three days after discharge and the decision was made to externalize the shunt pending completion of blood and wound culture. Prior to surgery, the patient was afebrile and blood serum chemistry was within normal limits. The patient was started on empiric antibiotics with vancomycin and cefepime. CSF analysis again failed to demonstrate white blood cells, red blood cells and glucose was elevated at 80 mg/dL. Initial cultures demonstrated acid-fast bacilli and growth on media implied it was a rapid grower mycobacterium. The patient was started on empiric treatment for atypical rapid growing mycobacterium with azithromycin, imipenem and zyvox. Serial CSF analyses were performed to ensure negative gram stain and culture prior to internalization of the VP shunt. Once sterilization of the CSF culture was complete and shunt was internalized, the patient was discharged home on levofloxacin and clarithromycin while awaiting final identification and sensitivity of the organism. The patient completed a total of 15 days of IV antibiotics before being transitioned to an oral regimen. Final identification illustrated *M. abscessus*.

## Discussion

Rapidly growing nontuberculous mycobacteria is a distinct class of nonpigmented mycobacteria that show growth on solid media within seven days. It is made up of about 40 recognized species, some examples include *M. abscessus*, *Mycobacterium chelonae* and *Mycobacterium fortuitum,* which are known to cause human infections [2]. They are aerobic, gram-positive rods that can produce a biofilm and may survive at temperatures of up to 45℃, and species such as *M. abscessus* have the capability of resisting the action of common household disinfectants like chlorine. Transmission from person to person has not been reported, rather cases are usually linked to soil or water contaminated wounds and exposure from contaminated equipment [4].

RGM may be found within the normal flora of the respiratory tract and as such, do not always indicate infection when seen in sputum or gastric samples. However, identification of the pathogen within a normally sterile site, such as CSF, is highly indicative of infection. Literature review points to 12 cases of *M. abscessus* causing meningitis, of which only two involve VP shunts [2]. The first case involving the infection of a VP shunt by the organism was reported in 2015 in a 39-year-old male patient with cerebral palsy who, like our patient, had not undergone surgical manipulation of the shunt for more than 10 years. The male patient struggled with an extensive history of gastrointestinal issues that eventually required the placement of a gastrostomy tube, believed to be the original source of infection. The management of the CNS infection involved nontuberculous antimicrobials as well as the replacement of the VP shunt. Recurrence of infection forced further treatment and full eradication was achieved only after a third ventriculostomy along with a prolonged regimen of systemic antimicrobials [2,5]. Evidence suggests that the pathogen’s ability to produce a biofilm explains its long latent period and makes infection resolution without removal of the infected foreign material very difficult [1].

The second known case was reported in a 59-year-old male that presented with a three-month history of cognitive decline and low-grade fevers. The patient’s medical history included a VP shunt placement secondary to tubercular meningitis with hydrocephalus further managed with a 15-month course of anti-tubercular drugs. The patient remained asymptomatic for 16 years, but in 2008 sustained multiple lacerations after an altercation that resulted in global deterioration with cognitive decline, upward gaze paresis and ataxic gait thereafter. Neurological evaluation after the reported three-month decline revealed raised intracranial tension with hydrocephalus. CSF analysis indicated possible ventriculitis and acid-fast staining revealed acid-fast bacilli later identified to be *M. abscessus*. The patient was treated with IV antibiotics, the VP shunt was removed and an external ventricular drainage was placed. Following a total of three sterile cultures, a VP shunt was reinserted, but the patient developed fevers and neurological decompensation one week after surgery. He did not respond to further treatment and unfortunately did not survive [1].

Our patient is the third reported case of infection caused by *M. abscessus* involving a VP shunt. Given the patient’s multiple comorbidities, a multidisciplinary team approach to treatment was held between neurosurgery, primary care and infectious disease. The review of the literature recommended using two or more antimicrobials that can penetrate the blood-brain barrier. As stated previously, the patient was started on a course of azithromycin, imipenem and zyvox before transitioning to clarithromycin and levofloxacin until final sensitivity was completed. Sequencing performed showed the RGM to be susceptible to amikacin, clarithromycin, imipenem and linezolid. The organism was resistant to ciprofloxacin, doxycycline, minocycline, moxifloxacin, Trimethoprim-sulfa and intermediate to cefoxitin. Because of the resistance pattern, levofloxacin was discontinued and linezolid was initiated. The patient was on this dual therapy regimen for three weeks and weekly labs were performed to ensure platelets counts were stable.

## Conclusions

This case demonstrates that physicians should have a high index of suspicion for atypical mycobacterium VP shunt infection in patients that present with subacute meningitis or present with damaged hardware. Atypical mycobacterium is known to be indolent and form biofilms on indwelling hardware. After initiation of anti-tuberculous chemotherapy, response to treatment should be evaluated clinically. Physicians should consider a prolonged course of treatment if complications occur.
